# Murine CD4 T Cells Produce a New Form of TGF-β as Measured by a Newly Developed TGF-β Bioassay

**DOI:** 10.1371/journal.pone.0018365

**Published:** 2011-04-11

**Authors:** Takatoku Oida, Howard L. Weiner

**Affiliations:** Center for Neurologic Diseases, Brigham and Women's Hospital, Harvard Medical School, Boston, Massachusetts, United States of America; University Paris Sud, France

## Abstract

**Background:**

It is generally assumed that T cells do not produce active TGF-β since active TGF-β as measured in supernatants by ELISA without acidification is usually not detectable. However, it is possible that active TGF-β from T cells may take a special form which is not detectable by ELISA.

**Methodology/Principal Findings:**

We constructed a TGF-β bioassay which can detect both soluble and membrane-bound forms of TGF-β from T cells. For this bioassay, 293T cells were transduced with (caga)_12_ Smad binding element-luciferase along with CD32 (Fc receptor) and CD86. The resulting cells act as artificial antigen presenting cells in the presence of anti-CD3 and produce luciferase in response to biologically active TGF-β. We co-cultured pre-activated murine CD4^+^CD25^−^ T cells or CD4^+^CD25^+^ T cells with the 293T-caga-Luc-CD32-CD86 reporter cells in the presence of anti-CD3 and IL-2. CD4^+^CD25^−^ T cells induced higher luciferase in the reporter cells than CD4^+^CD25^+^ T cells. This T cell-produced TGF-β is in a soluble form since T cell culture supernatants contained the TGF-β activity. The TGF-β activity was neutralized with an anti-mouse LAP mAb or an anti-latent TGF-β/pro-TGF-β mAb, but not with anti-active TGF-β Abs. An anti-mouse LAP mAb removed virtually all acid activatable latent TGF-β from the T cell culture supernatant, but not the ability to induce TGF-β signaling in the reporter cells. The induction of TGF-β signaling by T cell culture supernatants was cell type-specific.

**Conclusions/Significance:**

A newly developed 293T-caga-Luc-CD32-CD86 reporter cell bioassay demonstrated that murine CD4 T cells produce an unconventional form of TGF-β which can induce TGF-β signaling. This new form of TGF-β contains LAP as a component. Our finding of a new form of T cell-produced TGF-β and the newly developed TGF-β bioassay system will provide a new avenue to investigate T cell function of the immune system.

## Introduction

TGF-β is an immunoregulatory cytokine that controls immune responses by multiple mechanisms [1]. TGF-β-deficient mice manifest an autoimmune syndrome and do not survive longer than 3–4 wks after birth [2], [3]. Moreover, it has been shown that TGF-β initiates Th17 differentiation in combination with IL-6 or IL-21 [4], [5], [6], [7], [8]. Although IL-17 is a dominant factor in the induction of autoimmune diseases such as experimental autoimmune encephalomyelitis [9] and collagen-induced arthritis [10], IL-17 production is not seen in TGF-β_1_^−/−^ mice [5]. Although many cell types produce TGF-β, T cell-produced TGF-β is plays an important role in the control of autoimmune responses and Th17 differentiation. Thus, T cell-specific TGF-β conditional knockout mice develop fatal autoimmune disease even though they survive longer than TGF-β^−/−^ mice [11], and Th17 differentiation is hampered in these mice [11], indicating that TGF-β produced by T cells themselves is required for Th17 differentiation.

TGF-β is produced as a pro-form (pro-TGF-β), and is intracellularly processed by furin proprotein convertase into latent TGF-β. Latent TGF-β is a non-covalently associated complex consisting of latency-associated peptide (LAP) which is the N-terminal portion of pro-TGF-β, and mature TGF-β which is made of the C-terminal of pro-TGF-β. Latent TGF-β cannot bind TGF-β receptors and thus further activation processes are required for biological activity [12]. It is unknown how T cell-produced TGF-β is activated.

Murine T cell culture supernatants usually do not contain active TGF-β when measured by ELISA without acidification. Thus, it is generally believed that T cells do not produce active TGF-β.

Nakamura et al. [13] first reported that murine CD4^+^CD25^+^ regulatory T cells (Tregs) expressed surface LAP and/or TGF-β (LAP/TGF-β), and they proposed that the membrane-bound TGF-β mediated suppressive activity of Tregs. We also confirmed that Foxp3^+^ Tregs express surface LAP/TGF-β by using our newly developed anti-mouse LAP/TGF-β mAbs [14]. Human FOXP3^+^ Tregs have also been shown to express surface LAP [15], [16], [17]. It is possible that surface LAP/TGF-β on T cells can trigger TGF-β signaling in target cells by a cell-cell contact manner, give that active TGF-β is usually not detectable from T cell culture supernatants by ELISA. Alternatively, active TGF-β may be a rapidly-consumed, short-lived cytokine in T cell culture. Although there is no experimental evidence thus far, it is also possible that T cells produce biologically active TGF-β in a form that is not detectable by ELISA.

Given these possibilities, we developed a bioassay system which detects TGF-β activity, rather than the specific molecular form (the 25 kDa free TGF-β dimer) that an ELISA detects. This new bioassay consists of reporter cells that have direct contact with T cells and which can sense both short-lived and membrane-bound forms of TGF-β. 293T cells were transduced with a TGF-β reporter vector which has repeated the CAGA Smad binding elements in the promoter followed by luciferase, and with CD32 (Fc receptor) and CD86.

By using the newly developed 293T-caga-Luc-CD32-CD86 reporter cells, we found that pre-activated murine CD4 T cells induced high luciferase signals in the reporter cells. Although Foxp3^+^ T cells expressed surface LAP/TGF-β [14], pre-activated CD4^+^CD25^−^ T cells induced much higher TGF-β signal than pre-activated CD4^+^CD25^+^ T cells. The T cell-produced TGF-β was a soluble form since T cell culture supernatants contained TGF-β activity. The T cell-produced TGF-β is not the canonical 25 kDa mature TGF-β since a TGF-β ELISA did not detect the 25 kDa mature TGF-β form in the same T cell culture supernatants. Surprisingly, the TGF-β activity in T cell culture supernatants was neutralized with an anti-LAP mAb and with an anti-pro-TGF-β/latent TGF-β mAb, but not with anti-mature TGF-β Abs. TGF-β activity remained in culture supernatants even after the culture supernatant was treated with immobilized anti-LAP mAb by which latent TGF-β detected by TGF-β ELISA after acidification was virtually all depleted. Thus, T cell-produced TGF-β takes a unique molecular form which contains LAP as a component, and from which 25 kDa mature TGF-β is not released even after acidification. T cell-produced TGF-β initiated TGF-β signaling not in all cell types, suggesting that cell type-specific factors are required to sense the T cell-produced TGF-β.

## Results

### Generation of a TGF-β reporter cell line

**Figure 1:**
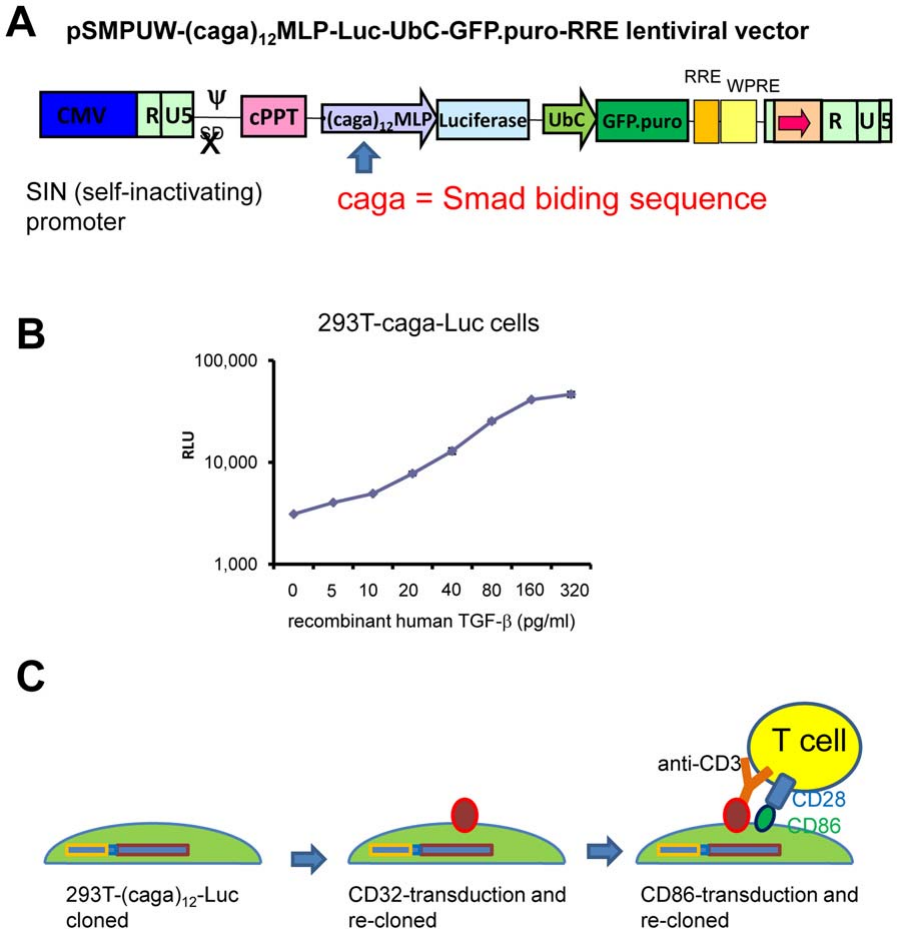
Generation of a TGF-β reporter cell line. (A) Schematic diagram of pSMPUW-(caga)_12_-Luc lentivirus based TGF-β reporter vector. (B) Representative luciferase response of 293T-caga cells to recombinant TGF-β. 293T cells were transduced with the pSMPUW-(caga)_12_-Luc vector, and cultured in the presence of recombinant human TGF-β for 16 hrs. Luciferase activity was measured from the cell lysates. Mean ± S.D. from duplicates are shown. (C) Schematic diagram of generation of 293T-caga-Luc-CD32-CD86 cells and how the cells function as artificial antigen presenting cells.

It has been reported that CAGA is a Smad binding element and a promoter assay vector containing tandem repeats of CAGA in the minimum promoter region ((caga)_12_-MLP-Luc) is a sensitive and specific TGF-β reporter vector [18], [19]. We inserted the (caga)_12_-MLP-Luc segment into a promoterless lentiviral vector (pSMPUW) to construct a lentivirus based universal TGF-β reporter vector (pSMPUW-(caga)_12_-MLP-Luc-UbC-EGFP.puro-RRE) (Figure 1A).

Human embryonic kidney 293T cells were then transduced with the TGF-β reporter vector (293T-caga-Luc cells). The response curve of 293T-caga-Luc cells to recombinant TGF-β was able to detect as little as 2 pg/ml recombinant human TGF-β (Figure 1B). To make the 293T-caga-Luc cells function as artificial antigen presenting cells, the 293T-caga-Luc cells were transduced with mouse CD32 (Fc receptor) and mouse CD86 retroviral vectors (Figure 1C). *Serpinb9* (granzyme B inhibitor) and *Serpinb9b* (granzyme M inhibitor) were also retrovirally expressed. These granzyme inhibitor genes suppressed CD4 T cell-mediated killing of CD32- and CD86-transduced mink lung epithelial cells (MLEC) (data not shown), and it is expected that the granzyme inhibitor genes protect 293T cells from CD4 T cell-mediated killing, too, although 293T cells are relatively resistant to CD4 cell-mediated killing without these gene transductions. The transduced 293T cells were cloned at each step and high responding or high expressing clones were selected. The resulting TGF-β reporter cell line is termed as 293T-caga-Luc-CD32-CD86.

### Detection of TGF-β activity from CD4 T cells in coculture

**Figure 2:**
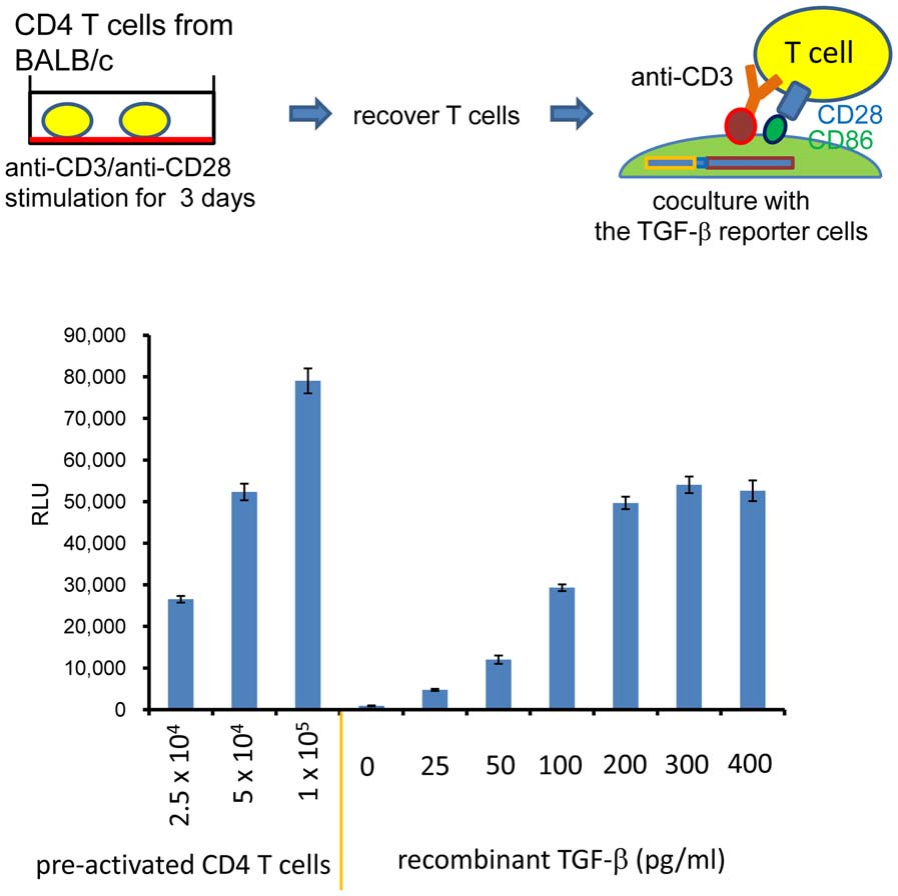
TGF-β bioassay in co-culture. Mouse CD4 T cells were stimulated with plate-bound anti-CD3/CD28 for 2 days, and rested for 1 day. The pre-activated CD4 T cells were recovered, and the indicated numbers of T cells were added to 293T-caga-Luc-CD32-CD86 cells with 0.5 µg/ml anti-CD3 antibody. Recombinant human TGF-β was also added as a standard. After 16 hr of culture, the reporter cells were lysed and the luciferase activity was measured. Error bars represent mean ± S.D. of duplicates.

We tested whether murine CD4 T cells have TGF-β activity by using the 293T-caga-Luc-CD32-CD86 TGF-β reporter cells. For these experiments we co-cultured T cells with 293T-caga-Luc-CD32-CD86 TGF-β reporter cells in presence of anti-CD3 antibody. In preliminary experiments, we found that freshly prepared CD4 T cells barely induced luciferase during 24 hr co-culture. Then, we tested pre-activated CD4 T cells. Thus, mouse CD4 T cells were stimulated with plate-bound anti-CD3/CD28 for two days, rested for one day and then co-cultured with 293T-cgga-Luc-CD32-CD86 TGF-β reporter cells with anti-CD3 for 16 hrs (Figure 2A). As shown Figure 2B, CD4 T cells induced high luciferase activity in a cell number dependent manner. Interestingly, although the response to recombinant TGF-β became saturated above 200 pg/ml, CD4 T cells often induced higher luciferase activity than the saturation point. It should be noted that the re-stimulation at this time point minimally induced activation-induced cell death, while re-stimulation at later time points induced notable cell death under microscopic observation. Higher luciferase signal was observed at later time points when anti-FasL mAb was added to block the activation-induced cell death (Figure S1). Thus, it is not likely that the TGF-β activity is generated from dying T cells.

### TGF-β activity from Tregs

**Figure 3:**
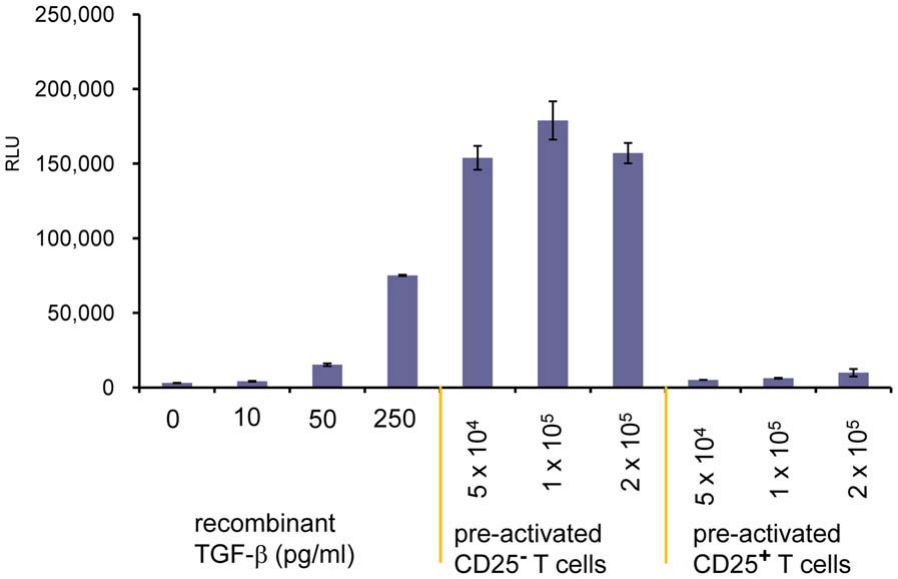
TGF-β bioassay of CD4^+^CD25^+^ Tregs. Mouse CD4^+^CD25^+^ T cells and CD4^+^CD25^−^ T cells were stimulated with plate-bound anti-CD3/CD28 for 2 days, and rested for 1 day in presence of 100 U/ml IL-2. These pre-activated CD4 T cells were recovered, and the indicated numbers of T cells were added to 293T-caga-Luc-CD32-CD86 cells with 0.5 µg/ml anti-CD3 antibody and 100 U/ml IL-2 for 16 hrs after which luciferase activity was measured. Error bars represent mean ± S.D. of duplicates.

We previously reported that activated Fox3^+^ CD4 T cells express surface LAP/TGF-β using our newly developed anti-mouse LAP/TGF-β mAbs [14]. We tested whether these surface LAP/TGF-β-expressing Foxp3^+^ CD4 T cells were CD4 T cells with TGF-β activity. CD4^+^CD25^+^ (>90% Foxp3^+^) cells and CD4^+^CD25^−^ (>98% Foxp3^−^) cells were isolated and stimulated with plate-bound anti-CD3/CD28 in the presence of IL-2 for two days and rested for one day in presence of IL-2. They were then co-cultured with 293T-caga-Luc-CD32-CD86 TGF-β reporter cells in presence of anti-CD3 and IL-2 for 16 hrs. The T cells have tight cell to cell contact with the reporter cells both by the CD32-anchored anti-CD3 Ab and by CD86; thus the reporter cells should be able to sense membrane-bound TGF-β activity if present. As shown in Figure 3, CD4^+^CD25^+^ T cells had less TGF-β activity in the co-culture bioassay than CD4^+^CD25^−^ T cells. These results demonstrate that non-Tregs are the main CD4 T cells with TGF-β activity by the reporter assay.

### No requirement for direct contact with T cells for induction of TGF-β signaling

**Figure 4:**
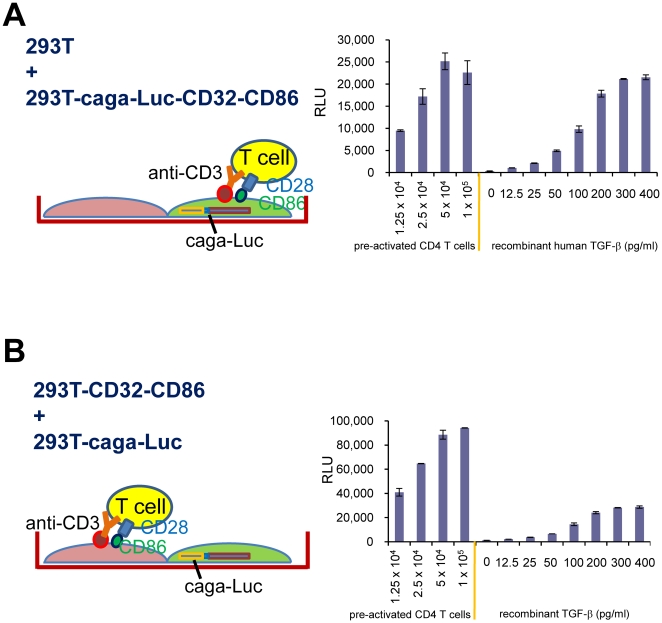
Investigation of requirement for direct contact between T cells and TGF-β reporter cells. (A) 293T-caga-Luc-CD32-CD86 cells were mixed with an equal number of un-manipulated 293T cells. Under this condition, a single 293T-caga-Luc-CD32-CD86 cell stimulates CD4 T cells and responds to TGF-β activity produced by the same T cells. (B) 293T-caga cells that did not have CD32 or CD86 were mixed with 293T-CD32-CD86 cells lacking the (caga)_12_-Luc reporter. Under this condition the 293T-caga reporter cells have minimal contact with CD4 T cells.

Although we initially anticipated that membrane-bound TGF-β on Foxp3^+^ CD4 T cells would trigger TGF-β signaling in the reporter cells, as described above, this was not the case. To further investigate the role of membrane-bound TGF-β, we asked whether direct contact between T cells and the reporter cells was required to initiate TGF-β signaling. In order to address this question, 293T-caga-Luc cells that did not have surface CD32 or CD86 were mixed with CD32- and CD86-trandsduced 293T cells that did not have the (caga)_12_-Luc reporter (Figure 4B). If T-reporter contact was required to present T cell-produced TGF-β to TGF-β receptors on the reporter cells, there would be diminished induction of luciferase in this condition. We found, however, that pre-activated CD4 T cells stimulated with 293T-CD32-CD86 cells in presence of anti-CD3 induced TGF-β signaling in 293T-caga-Luc cells as well as or better than the condition in which 293T-caga-Luc-CD32-CD86 cells were used as reporter cells (Figure 4A). This result indicates that the T cell-produced TGF-β that results in TGF-β activity as measured in our reported system is soluble and can diffuse to adjacent cells.

### TGF-β activity in T cell culture supernatants

**Figure 5:**
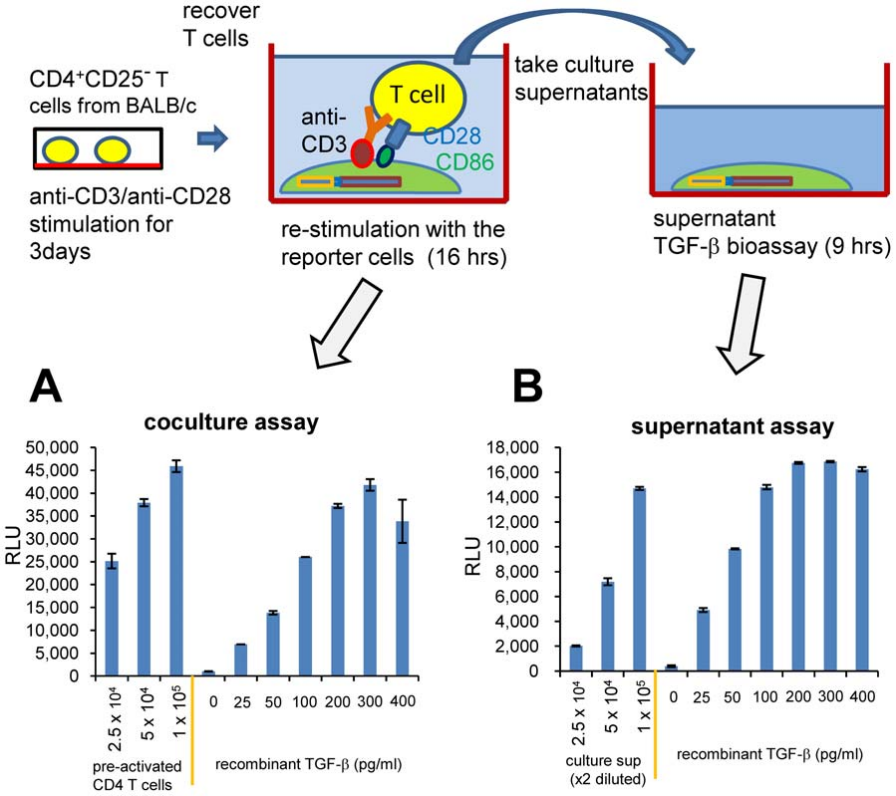
TGF-β bioassay from T cell culture supernatants. (A) Pre-activated CD4^+^CD25^−^ T cells were re-stimulated with 293T-caga-Luc-CD32-CD86 cells in the presence of anti-CD3 as a co-culture assay. (B) Supernatants were taken from the co-culture assay, added to new wells of 293T-caga-Luc-CD32-CD86 reporter cells and luciferase activity was measured. Error bars represent mean ± S.D. of duplicates.

After demonstrating that T cell-produced TGF-β is in a soluble form, we next asked whether this form accumulates in culture supernatants. To address this, pre-activated CD4^+^CD25^−^ T cells (anti-CD3/28 for 3 days) were re-stimulated with the reporter cells plus anti-CD3 for 16 hrs as the co-culture assay (Figure 5A), and the culture supernatants were also taken at the end of the assay. The culture supernatants were added to fresh 293T-caga-Luc-CD32-CD86 TGF-β reporter cells (without live T cells), and the reporter cells were cultured for 9 hrs (Figure 5B). We found that the T cell culture supernatant from the 1×10^5^ cells/well induced luciferase activity equivalent to approximately 200 pg/ml of recombinant human TGF-β. Thus, TGF-β activity accumulates in T cell culture supernatants. We also measured the same culture supernatant by TGF-β ELISA without acidification which detects the 25 kDa free TGF-β dimer and found that the amount of active TGF-β as measured by the TGF-β ELISA was 10 pg/ml. “Total” TGF-β as measured by ELISA after acidification was found to be 486 pg/ml (Table 1). These results indicate that the T cell-produced TGF-β activity takes an unconventional form which is different from the 25 kDa free TGF-β dimer and is not detectable by ELISA.

**Table 1:** TGF-β in a T cell culture supernatant was measured by the 293T-caga-CD32-CD86-caga bioassay (TGF-β activity), by ELISA without acidification (the 25 kDa free TGF-β dimer), and by ELISA after acidification ( “total TGF-β”).

TGF-β in culture supernatant		
293T-(caga)_12_-Luc	ELISA	ELISA
bioassay	without acidification	after acidification
(activity)	(25 kDa free dimer)	(“total”)
∼200 pg/ml	10 pg/ml	486 pg/ml

### T cell-produced TGF-β activity induces Smad2 phosphorylation in 293T-caga-Luc-CD32-CD86 cells

**Figure 6:**
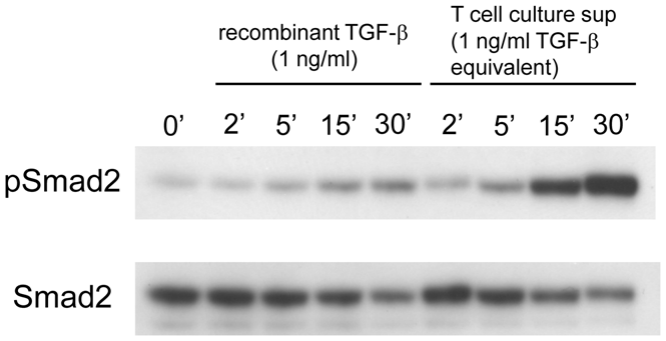
Smad2 phosphorylation by T cell culture supernatants. Recombinant TGF-β (1 ng/ml) or a T cell culture supernatant containing equivalent amount of TGF-β activity was added to 293T-caga-Luc-CD32-CD86 cells. After 2, 5, 15, and 30 min, the cells were lysed, and the lysates were run on SDS-PAGE. After transfer to a PVDF membrane, the membrane was blotted with anti-phospho-Smad2 Ab, and reblotted with anti-Smad2/3 Ab.

To confirm that the luciferase activity was a consequence of TGF-β signaling, we determined Smad2 phosphorylation in 293T-caga-Luc-CD32-CD86 cells after exposure to T cell culture supernatants. T cell culture supernatants from anti-CD3/CD28-stimulated cultures of pre-activated CD4^+^CD25^−^ T cells contained higher TGF-β activity (2–3 ng/ml recombinant TGF-β equivalent) than cultures re-stimulated with 293T-caga-Luc-CD32-CD86 reporter cells/anti-CD3 (data not shown). Thus, we used culture supernatants from plate-bound anti-CD3/CD28 re-stimulated CD4^+^CD25^−^ T cells for further studies. 1 ng/ml recombinant human TGF-β or a diluted T cell culture supernatant adjusted to the equivalent TGF-β activity determined by the 293T-caga-Luc-CD32-CD86 TGF-β bioassay was added to 293T-caga-Luc-CD32-CD86 cells for the indicated time, and the cell lysates were run on SDS-PAGE and blotted with anti-phospho-Smad2 antibody. Although the T cell culture supernatant was diluted to normalize the TGF-β activity, the T cell culture supernatant induced stronger Smad2 phosphorylation than recombinant TGF-β at all the time points (Figure 6). This result suggests that T cell-produced TGF-β activity is qualitatively different from the 25 kDa free TGF-β dimer.

### T cell-produced TGF-β activity is not found in the ELISA detectable “total” TGF-β fraction

**Figure 7:**
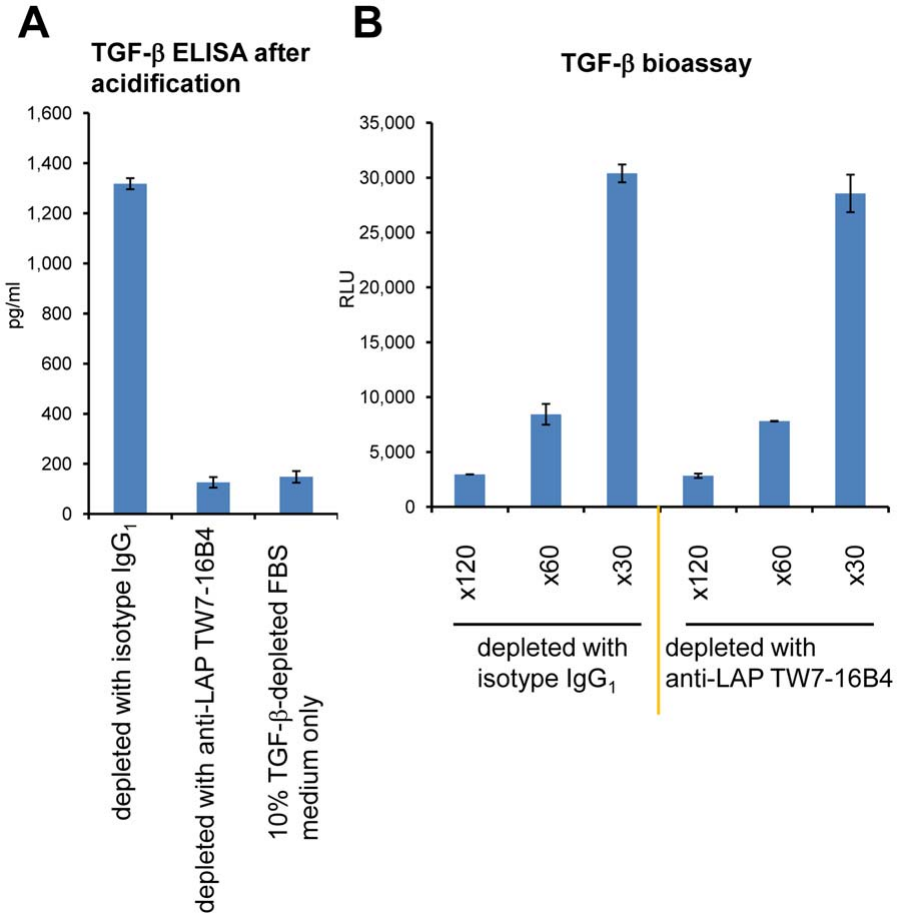
Immunologic depletion of “total” TGF-β from culture supernatants. (A) A T cell culture supernatant from plate-bound anti-CD3/CD28 re-stimulated CD4^+^CD25^−^ T cells cultured in 10% TGF-β-depleted FBS IMDM was treated with control IgG_1_ mAb-coated magnetic beads or anti-mouse LAP TW7-16B4 mAb-coated magnetic beads. The remaining “total” TGF-β in the culture supernatant was measured by TGF-β ELISA after acidification. Error bars represent mean ± S.D. of duplicates. (B) TGF-β activity in the same control IgG_1_-treated or anti-LAP TW7-16B4-treated T cell culture supernatant was measured by the 293T-caga-Luc-CD32-CD86 bioassay after ×30, ×60 and ×120 dilutions.

TGF-β measured by ELISA after acidification is termed “total” TGF-β. This is based on the assumption that following acidification, the 25 kDa free TGF-β dimer is released from any form of TGF-β, such as the small latent TGF-β complex and the large latent TGF-β complexes. We thus asked whether T-cell produced TGF-β activity resides in the “total” TGF-β fraction. Since FBS-derived latent TGF-β affects TGF-β ELISA after acidification, we used TGF-β-depleted FBS for the culture medium [20]. The background bovine TGF-β remaining in the 10% TGF-β-depleted FBS-supplemented medium was 148 pg/ml. We treated a T cell culture supernatant with anti-LAP mAb TW7-16B4-coupled magnetic beads (or an IgG_1_ isotype control mAb MOPC21) to deplete latent TGF-β in the supernatant. The amount of “total” TGF-β detected by ELISA after acidification in control MOPC21-treated T cell culture supernatant was 1,318 pg/ml, whereas the anti-LAP TW7-16B4-treated T cell culture supernatant contained 126 pg/ml “total” TGF-β (Figure 7A). Since anti-murine LAP TW7-16B4 mAb does not cross-react with bovine LAP (data not shown), we conclude that all T cell-derived “total” TGF-β was removed by the TW7-16B4-coupled magnetic bead treatment. However, TGF-β activity in the TW7-16B4-treated T cell culture supernatant was intact since the anti-LAP TW7-16B4-treated T cell culture supernatant induced an identical TGF-β response in 293T-caga-Luc-CD32-CD86 reporter cells as the control MOPC21-treated T cell culture supernatant (Figure 7B). These data indicate that T cell-produced TGF-β activity is not contained in the ELISA detectable “total” TGF-β fraction.

### Neutralization of T cell-produced TGF-β activity with anti-LAP/latent TGF-β mAbs

**Figure 8:**
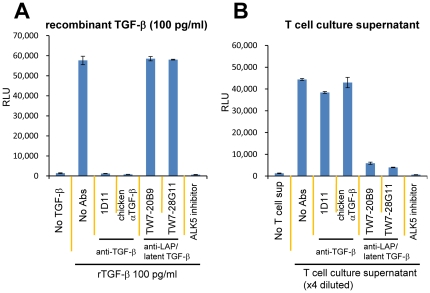
Effects of anti-LAP or anti-latent TGF-β/pro-TGF-β mAbs on TGF-β activity in T cell culture supernatants. (A) Recombinant human TGF-β was pre-mixed with anti-active TGF-β Abs (1D11 or chicken anti-TGF-β), an anti-LAP mAb (TW7-20B9), an anti-latent TGF-β/pro-TGF-β mAb (TW7-28G11) (final 50 µg/ml), or ALK5 inhibitor II (final 1 µM), and then added to 293T-caga-Luc-CD32-CD86 reporter cells. (B) A T cell culture supernatant containing an equivalent amount of TGF-β was pre-mixed with the indicated Abs or the ALK5 inhibitor, and added to 293T-caga-Luc-CD32-CD86 cells. The luciferase activity was measured after 16 hr culture. Error bars represent mean ± S.D. of duplicates.

Since our results above indicate that T cell-produced TGF-β activity takes a form that is different from the 25 kDa free TGF-β dimer, we tested anti-active TGF-β Abs and anti-mouse LAP/latent TGF-β mAbs to better understand the structure of T cell-produced TGF-β. Recombinant TGF-β (100 pg/ml) and a T cell culture supernatant diluted to the equivalent activity were treated with 50 µg/ml of anti-TGF-β Abs (1D11 or chicken polyclonal anti-TGF-β), anti-mouse LAP mAb (TW7-20B9), or anti-latent TGF-β/pro-TGF-β mAb (TW7-28G11), and then tested for TGF-β activity by the 293T-caga-Luc-CD32-CD86 bioassay. As expected anti-TGF-β Abs neutralized recombinant TGF-β activity, whereas anti-LAP/anti-latent TGF-β/pro-TGF-β mAbs did not (Figure 8A). Surprisingly, anti-TGF-β Abs did not neutralize TGF-β activity in the T cell culture supernatant (Figure 8B). The ALK5 inhibitor II [14] completely blocked luciferase induction by the T cell culture supernatant (Figure 8B), confirming that the luciferase production was downstream of the TGF-β signaling. On the other hand, anti-LAP mAb TW7-20B9 and anti-latent TGF-β/pro-TGF-β TW7-28G11 inhibited TGF-β activity in the culture supernatant (Figure 8B). Thus, T-cell produced TGF-β activity as measured by the 293T-caga-Luc-CD32-CD86 bioassay takes a molecular form that contains LAP as a component. Since anti-LAP mAb TW7-20B9 does not cross-react with human LAP (Figure S2), the new form of active TGF-β is truly produced from murine CD4 T cells, but not from the human 293T reporter cells.

### T cell-produced TGF-β is not latent TGF-β

**Figure 9:**
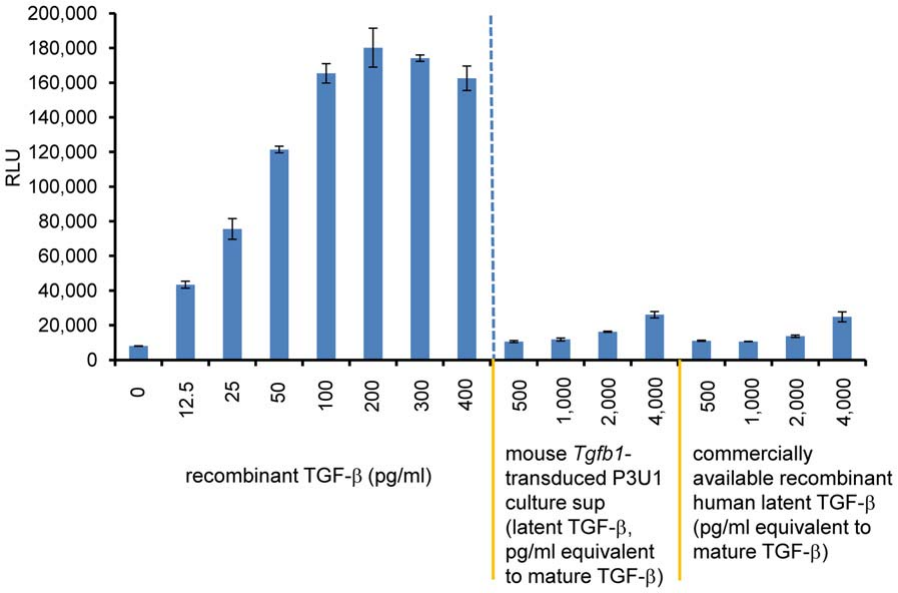
Response of 293T-caga-Luc-CD32-CD86 cells to latent TGF-β. Recombinant TGF-β, commercially available recombinant human latent TGF-β or a diluted culture supernatant of mouse *Tgfb1*-transduced P3U1 cells was added to 293T-caga-Luc-CD32-CD86 cells and the resultant luciferase activity was measured after 11 hrs. Error bars represent mean ± S.D. of duplicates.

Since the T cell-produced TGF-β contains LAP, it may simply represent the latent TGF-β complex and the 293T-caga-Luc-CD32-CD86 reporter cells have a TGF-β activation machinery which can initiate TGF-β signaling in response to latent TGF-β. To exclude this possibility, we added a commercially available recombinant human latent TGF-β or a culture supernatant of mouse TGF-β-transduced P3U1 cells to the 293T-caga-Luc-CD32-CD86 reporter cells. We found that mouse TGF-β-transduced P3U1 cells produced high amounts of latent TGF-β, as judged by a TGF-β ELISA with or without acidification. However, both latent TGF-β samples barely induced TGF-β signaling in the 293T-caga-Luc-CD32-CD86 cells (Figure 9). Even at 4,000 pg/ml, recombinant human latent TGF-β and mouse latent TGF-β-containing supernatants induced only faint luciferase signals (equivalent to less than 10 pg/ml recombinant TGF-β), which is likely explained by the presence of contaminating active TGF-β which was detected by ELISA without acidification (data not shown).

### Cell type-specific responses to T cell-produced TGF-β

**Figure 10:**
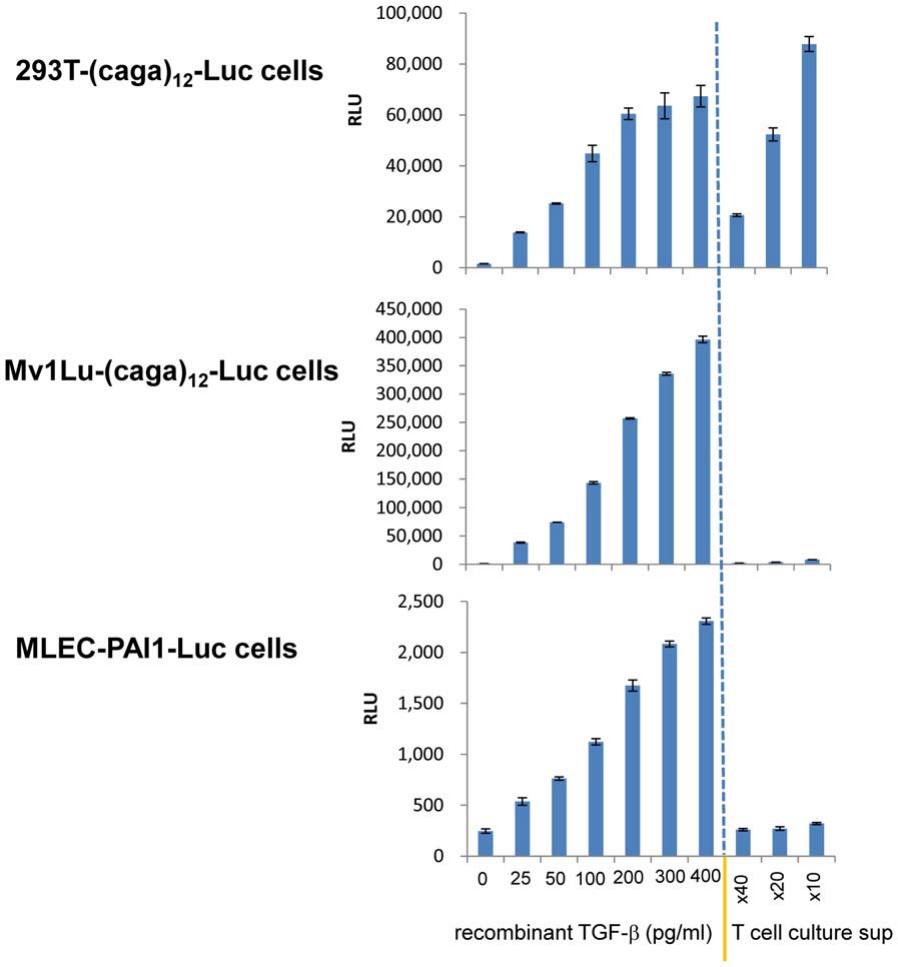
Cell type-specific responses to T cell-produced TGF-β. 293T-caga-Luc-CD32-CD86 cells (top), Mv1Lu-(caga)_12_-Luc cells (middle), or MLEC-PAI-1-Luc cells (bottom) were exposed to recombinant TGF-β or diluted T cell culture supernatants. The luciferase activity was measured after 11 hr culture. Error bars represent mean ± S.D. of duplicates.

Since T cell-produced TGF-β activity takes an unconventional TGF-β form, it is possible that it requires specific sensing machineries and that only certain cell types respond to T cell-produced TGF-β. To test this, we assayed T cell culture supernatants on other TGF-β reporter cell lines. The Mv1Lu-(caga)_12_-Luc cell line has the same the (caga)_12_-Luc reporter as the 293T-caga-Luc-CD32-CD86 cell line, and responds to recombinant TGF-β in a dose-dependent manner. However, we found that Mv1Lu-(caga)_12_-Luc cells did not respond to T cell culture supernatants (Figure 10, middle). Another well-known TGF-β bioassay reporter line is the mink lung epithelial cell (MLEC)-PAI-1-Luc cell line which has a PAI-1 promoter-driven luciferase reporter [21]; we found that MLEC-PAI-1-Luc cells also did not respond to T cell-produced TGF-β (Figure 10, bottom). These results indicate that T cell-produced TGF-β does not initiate TGF-β signaling in all cell types, but it requires cell type-specific molecular machineries to initiate TGF-β signaling.

## Discussion

We have previously reported [22] a TGF-β bioassay for T cells using the MLEC-PAI-1-Luc cell line, in which T cells were cocultured with reporter cells and stimulated with anti-CD3/CD28-coated beads attached to the reporter cell surface. This bioassay demonstrated that CD4^+^CD25^+^ Tregs produced active TGF-β which was neutralized with anti-TGF-β mAb 1D11. In order to improve on this bioassay, we first modified the bioassay so that the reporter cells behaved as artificial antigen presenting cells by transducing MLEC-PAI-1-Luc cells with mouse CD32 (Fc receptor) for anti-CD3 capture and with mouse CD86 for costimulation. Preliminary experiments showed that these CD32/CD86-transduced MLEC-PAI-1-Luc cells were killed by murine pre-activated CD4 T cells when T cells were co-cultured with the reporter cells in the presence of anti-CD3. Thus, we chose 293T cell line as TGF-β reporter cells as this cell line is resistant to CD4-mediated killing. We made a lentivirus based TGF-β reporter vector which contains repeated CAGA Smad binding elements in the promoter region linked luciferase (pSMPUW-(caga)_12_-Luc). 293T cells were transduced with (caga)_12_-Luc, mouse CD32 and mouse CD86. *Serpinb9* and *Serpinb9b* were also transduced as we found in preliminary experiments that these granzyme inhibitor genes acted to lessen CD4-mediated killing in MLEC-PAI-1-Luc cells.

When we used this new 293T-caga-Luc-CD32-CD86 reporter cell to assay activated T cells, we found that activated T cells produced high TGF-β activity in the 293T-caga-Luc-CD32-CD86 reporter cell assay even though we could not detect active TGF-β production by ELISA. Furthermore, we found that activated Foxp3^+^ CD4 T cells that express surface LAP/TGF-β [14] had lower TGF-β activity in the 293T-caga-Luc-CD32-CD86 reporter cell assay than activated CD25^−^ T cells. This TGF-β activity was detected in T cell culture supernatants indicating that the T cell-produced TGF-β was in the soluble form and was not membrane-bound. These findings are in contrast to our previous observations using MLEC-PAI-1-Luc cells [22] that when compared to CD4^+^CD25^−^ T cells, activated CD4^+^CD25^+^ T cells had higher TGF-β activity and this activity was linked to membrane bound TGF-β. We believe that this difference is because the T cell-produced TGF-β activity we observed using the 293T-caga-Luc-CD32-CD86 reporter cell assay consists of an unconventional form of TGF-β which can be measured by the 293T-caga reporter cells but not by MLEC-PAI-1-Luc cells. Thus it appears that Foxp3^+^ Tregs produce the canonical form of mature TGF-β which can be neutralized with anti-TGF-β mAb 1D11, whereas Foxp3^−^ non-Tregs produce a new form of TGF-β which we have identified with our new reporter assay and which can induce TGF-β signaling in a cell type-specific manner.

Whether all activated CD4^+^CD25^−^ T cells produce this newly described form of TGF-β or it is produced by a subset of CD4^+^CD25^−^ cells is an important question. In addressing this question, we found that CD4 T cells that produce this new form of TGF-β reside primarily in the CD62L^−^CD44^hi^ memory fraction as opposed to the CD62L^+^CD44^lo^ naive fraction (Figure S3). Whether differentiated Th cell subsets such as Th1, Th2, Th17, or Tr1 cells preferentially produce this form of TGF-β is an interesting future question.

The molecular structure and/or composition of this new form of TGF-β are unknown. It contains LAP as a component since the TGF-β activity measured by the 293T-caga-Luc-CD32-CD86 reporter cell is neutralized by an anti-LAP mAb and by an anti-latent TGF-β/pro-TGF-β mAb. Since mature TGF-β is not released from the new form of TGF-β by acidification, this suggests strong binding of the LAP segment to the TGF-β segment. This may be because LAP is linked to TGF-β by covalent bonding. Alternatively, disulfide bonding inside mature TGF-β may take irregular forms that make the TGF-β segment undetectable by ELISA even if the TGF-β segment is released by acidification. Whatever the case, it should be noted that one cannot detect the new form of TGF-β by ELISA even after acidification. Thus, this new form of TGF-β does not appear to be part of the “total” TGF-β detected by ELISA after acidification.

At this time we do not know what cell properties and/or molecules are required to initiate TGF-β signaling by the new form of TGF-β we have identified. In preliminary experiments, we did not find that T cell-produced TGF-β induced Smad2 phosphorylation in CD4 T cells or in bone marrow-derived DCs. However, it is possible that sub-populations of T cells or DCs would respond T cell-produced TGF-β under special conditions.

In conclusion, our work demonstrates that murine CD4 T cells produce an unconventional form of TGF-β which has biological activity as measured by 293T-caga-Luc-CD32-CD86 reporter cells but not by other assay systems and is not produced in significant amounts by conventional Treg cells. Our finding of a new form of T cell-produced TGF-β and the newly developed TGF-β bioassay system will provide a new avenue to investigate T cell function of the immune system.

## Materials and Methods

### Cell lines, Plasmids, and antibodies

The (caga)_12_-MLP-Luc vector was kindly provided from Dr. D. Vivien (the Universite' de Caen, Daix, France). Mv1Lu cells (ATCC) were stably transfected with the (caga)_12_-MLP-Luc plasmid (Mv1Lu-(caga)_12_-Luc cells). The mink lung epithelial cell line transfected with the Smad-responsive plasminogen activator inhibitor-1 promoter driving a luciferase reporter gene (MLEC-PAI-1-Luc) (originally developed by Abe et al. [21]) was obtained from Dr. L. van de Water (Massachusetts General Hospital, Boston, MA, USA). A lentivirus based TGF-β reporter vector was constructed by inserting the (caga)_12_-MLP-Luc segment into a promoterless lentiviral vector pSMPUW (Cell Biolabs) along with ubiquitin C promoter-driven a GFP-puro fusion gene as a selection marker (pSMPUW-(caga)_12_-Luc). pMCs retroviral vector was kindly provided Dr. T. Kitamura (Tokyo Univ., Tokyo, Japan) and pBMN retroviral vector was from Addgene under a MTA with Stanford University (Stanford, CA). A lentivirus supernatant and retrovirus supernatants were produced as described previously [23]. Human embryonic kidney 293T cells (Clontech) were sequentially transduced with pSMPUW-(caga)_12_-Luc, pBMN-mouse CD32 (without IRES), pBMN-mouse CD86 (without IRES), pMCs-*Serpinb9*-IRES-Thy1.1, and pMCs-*Serpinb9b*-IRES-Thy1.2 vectors with cloning in each step. The resultant cells were termed 293T-caga-Luc-CD32-CD86 cells. Anti-TGF-β hybridoma 1D11 was from ATCC, and chicken anti-TGF-β (AF-101-NA) was from R&D Systems. Anti-mouse LAP mAbs TW7-16B4 and TW7-20B9, and anti-latent TGF-β/pro-TGF-β mAb TW7-28G11 were described previously [14]. Anti-FasL (clone MFL3) was from BioLegend. ALK5 inhibitor II was from EMD/Calbiochem.

### CD4 T cell preparation and stimulation

Mice were housed in a pathogen-free environment and the animal protocols were approved by the Committee on Animals of Harvard Medical School (Harvard Medical Area Standing Committee on Animals, Protocol No. 02683). CD4 T cells were separated from BALB/c mice (The Jackson Laboratories) using a MACS CD4 purification kit (Miltenyi Biotec). When CD4^+^CD25^−^ T cells were prepared, biotinylated anti-CD25 antibody (7D4, BD Biosciences) was additionally mixed to the MCAS antibody cocktail. CD4^+^CD25^+^ T cells were prepared from the CD4 fraction by staining CD25-FITC (7D4, BD Biosciences) followed by anti-FITC MACS beads (Miltenyi Biotec), and by MS column separation. T cells were stimulated with plate-bound anti-CD3 and anti-CD28 (5 µg/ul each) for 2 days in 10% FBS-supplemented IMDM. In case of CD4^+^CD25^+^ T cell stimulation, 100 U/ml recombinant IL-2 was added both in CD4^+^CD25^+^ T cell cultures and in CD4^+^CD25^−^ T cell cultures. The cells were split into non-coated wells and rested for 1 day. The cells were recovered, washed with the culture medium, counted, and used for the TGF-β bioassay, or re-stimulated with plate-bound anti-CD3/CD28 for 24 h hrs for culture supernatants. When indicated, TGF-β-depleted FBS [20] was used for the culture medium.

### TGF-β ELISA

TGF-β ELISA was performed using anti-TGF-β mAb 1D11 as a coating antibody and biotinylated chicken anti-TGF-β IgY (BAF240, R&D Systems) as a detection antibody. Recombinant human TGF-β (R&D Systems) was used as a standard (0–2,000 pg/ml). Sample acidification was done by adding 1/10 volume of 1 N HCl, incubating at room temperature for 10 min, and neutralizing with 1/10 volume of 1 N NaOH/0.1 M Tris. The samples were then diluted twofold by adding 25 mM Tris buffered saline.

### TGF-β bioassay

293T-caga-Luc-CD32-CD86 TGF-β reporter cells were seeded at 2×10^4^ cells/100 µl/well on collagen-coated 96-well plates 1 day before addition of T cells. On the day of assay, 10 µl of 1 mg/ml anti-CD3 (145-2C11) (BD Biosciences) was added to each well (final 0.5 µg/ml). When CD4^+^CD25^+^ T cells were tested, IL-2 was also added to final 100 U/ml. Pre-activated CD4 T cells or recombinant human TGF-β (R&D Systems) were added at 100 µl/well (final total volume 210 µl/well) and cultured for 16 hrs. Similarly, the TGF-β bioassay from culture supernatants was conducted by adding 100 µl of diluted T cell culture supernatants to 293T-caga-Luc-CD32-CD86 cell culture wells and the reporter cells were cultured for the indicated time. When neutralizing antibodies were tested, T cell culture supernatants were diluted to be equivalent to 200 pg/ml recombinant TGF-β activity and were then premixed with the antibodies at 100 µg/ml for 30 min at room temperature. 100 µl of the mixture was added to 100 µl of 293T-caga-Luc-CD32-CD86 reporter wells. 293T-caga-Luc-CD32-CD86 cells were lysed with Glo-Lysis buffer (Promega), and luciferase activity was measured by ONE-Glo luciferease assay reagent (Promega).

### Detection of Smad2 phosphorylation

293T-caga-Luc-CD32-CD86 cells were exposed to recombinant human TGF-β (1 ng/ml) or a T cell culture supernatant diluted by TGF-β activity determined by the 293T-caga-Luc-CD32-CD86 bioassay equivalent to 1 ng/ml TGF-β. After 2, 5, 15, and 30 min, the cells were lysed with 1% Triton X-100, 0.25% deoxycholate, 0.1% SDS, 1 mM NaVO_4_, protease inhibitor cocktail (Pierce/Thermo), 25 mM Tris buffered saline. The lysates were clarified by centrifugation at 13,000 rpm for 15 min, and run on SDS-PAGE under reducing conditions. Western blot was conducted with rabbit anti-phospho-Smad2(Ser465/467) antibody (Cell Signaling) and with rabbit anti-Smad2/3 antibody (Cell Signaling).

### Depletion of “total” TGF-β from culture supernatants with an anti-LAP Ab

400 µl of 1 mg/ml of anti-mouse IgG magnetic beads (BioMag Plus, Polysciences) was placed in a 1.5 ml microcentrifuge tube and the beads were washed with PBS three times. 400 µl PBS containing 20 µg of anti-mouse LAP mAb TW7-16B4 or isotype control MOPC21 was added to the beads and incubated with rotation for 4 hrs. After washing with PBS three times, 0.6 ml of the T cell culture supernatant from plate-bound anti-CD3/CD28 re-stimulated CD4^+^CD25^−^ T cells in 10% TGF-β-depleted FBS IMDM was added to the bead pellets, and incubated at 4°C for overnight. The unbound supernatant was recovered by magnetic separation. The amount of TGF-β before and after separation was measured by TGF-β ELISA after acidification.

### Latent TGF-β

Recombinant human latent TGF-β was purchased from R&D Systems. Recombinant mouse latent TGF-β was made as a culture supernatant of mouse *Tgfb1*-transduced P3U1 cells. The 1× culture supernatant contained 84 ng/ml total TGF-β (mostly latent TGF-β) and 142 pg/ml active TGF-β determined by TGF-β ELISA with, and without acidification, respectively.

## Supporting Information

Figure S1 **Effect of blocking anti-Fas Ligand antibody to T cell-produced TGF- β.** Pre-activated CD4 T cells were harvested on day 4, which is one day delayed compared with the regular stimulation (day 3 recovery), and the CD4 T cells were co-cultured with 293T-caga-Luc-CD32-CD86 reporter cells in presence of blocking anti-FasL mAb.
(TIF)

Figure S2 **Species specificity of TW7 antibodies.** human *TGFB1*-transduced P3U1 cells (clone 32, without IRES-GFP) (GFP^−^ population) and mouse *Tgfb1*-transduced P3U1 cells (clone 11, containing IRES-GFP) (GFP^+^ population) were mixed and surface stained with TW7-16B4, TW7-20B9, or TW7-28G11 mAb. TW7-16B4 and TW7-20B9 stained only mouse *Tgfb1*-transduced cells while TW7-28G11 stained both human *TGFB1*-transduced cells and mouse *Tgfb1*-transduced cells.
(TIF)

Figure S3 **Production of TGF-β activity from naïve CD4 T cells and memory CD4 T cells.** CD62L^+^CD44^lo^ naïve CD4 T cells or CD62L^lo^CD44^hi^ memory CD4 T cells were stimulated with plate-bound anti-CD3/CD28 for 2 days, and rested for 1 day. The pre-activated CD4 T cells were recovered, and the indicated numbers of T cells were added to 293T-caga-Luc-CD32-CD86 cells with 0.5 µg/ml of anti-CD3 antibody. Recombinant human TGF-β was also added as a standard. After 16 hr culture, the reporter cells were lysed and the luciferase activity was measured. Error bars represent mean ± S.D. of duplicates.
(TIF)
