# Supplementary figures and images for: Murine CD4 T Cells Produce a New Form of TGF-β as Measured by a Newly Developed TGF-β Bioassay
Source: PLoS One. 2011 Apr 11;6(4):e18365. doi: 10.1371/journal.pone.0018365 (PMC3073999; doi:10.1371/journal.pone.0018365)

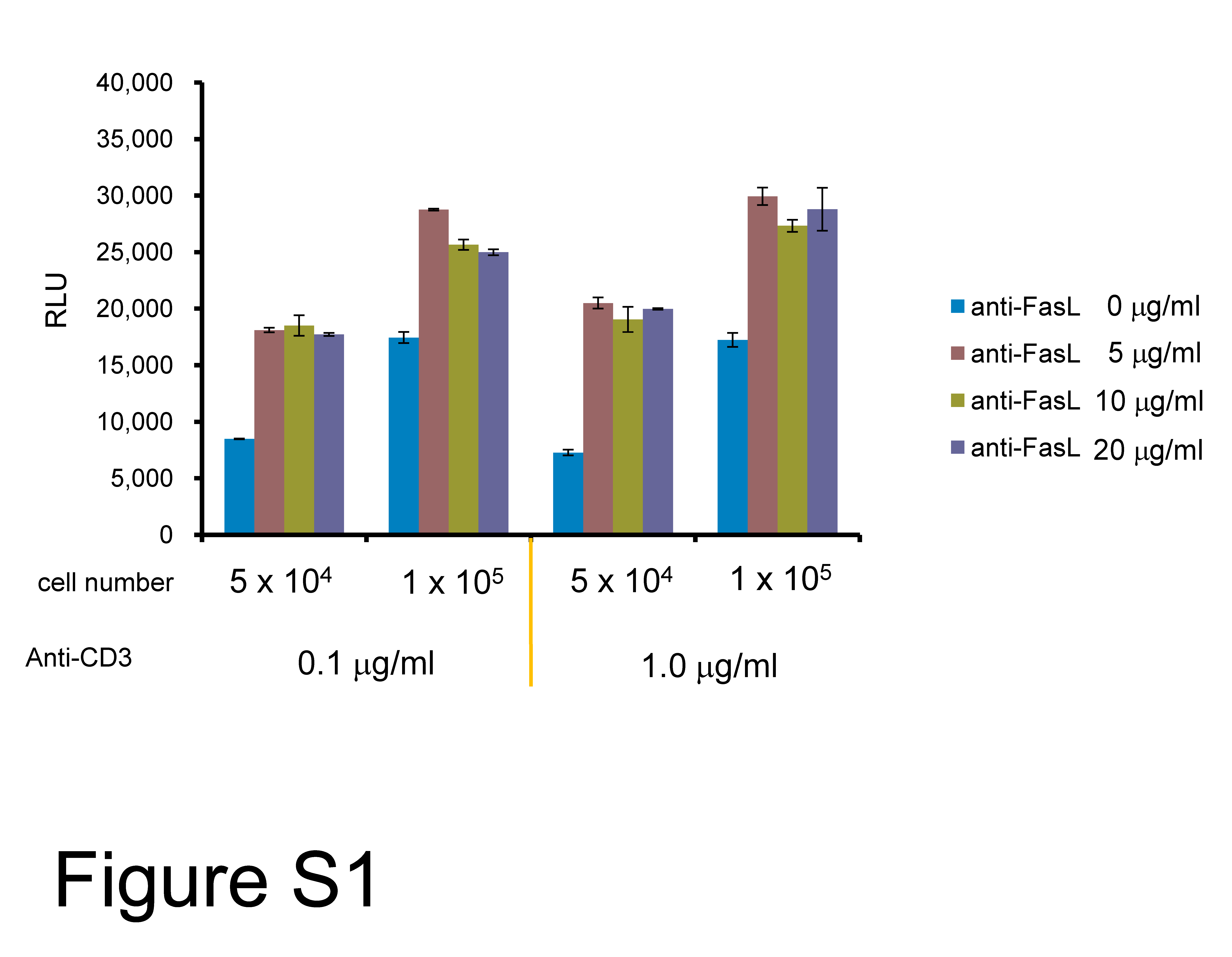

Supplement: Figure S1 — Effect of blocking anti-Fas Ligand antibody to T cell-produced TGF- β. Pre-activated CD4 T cells were harvested on day 4, which is one day delayed compared with the regular stimulation (day 3 recovery), and the CD4 T cells were co-cultured with 293T-caga-Luc-CD32-CD86 reporter cells in presence of blocking anti-FasL mAb. (TIF) [file pone.0018365.s001.tif]

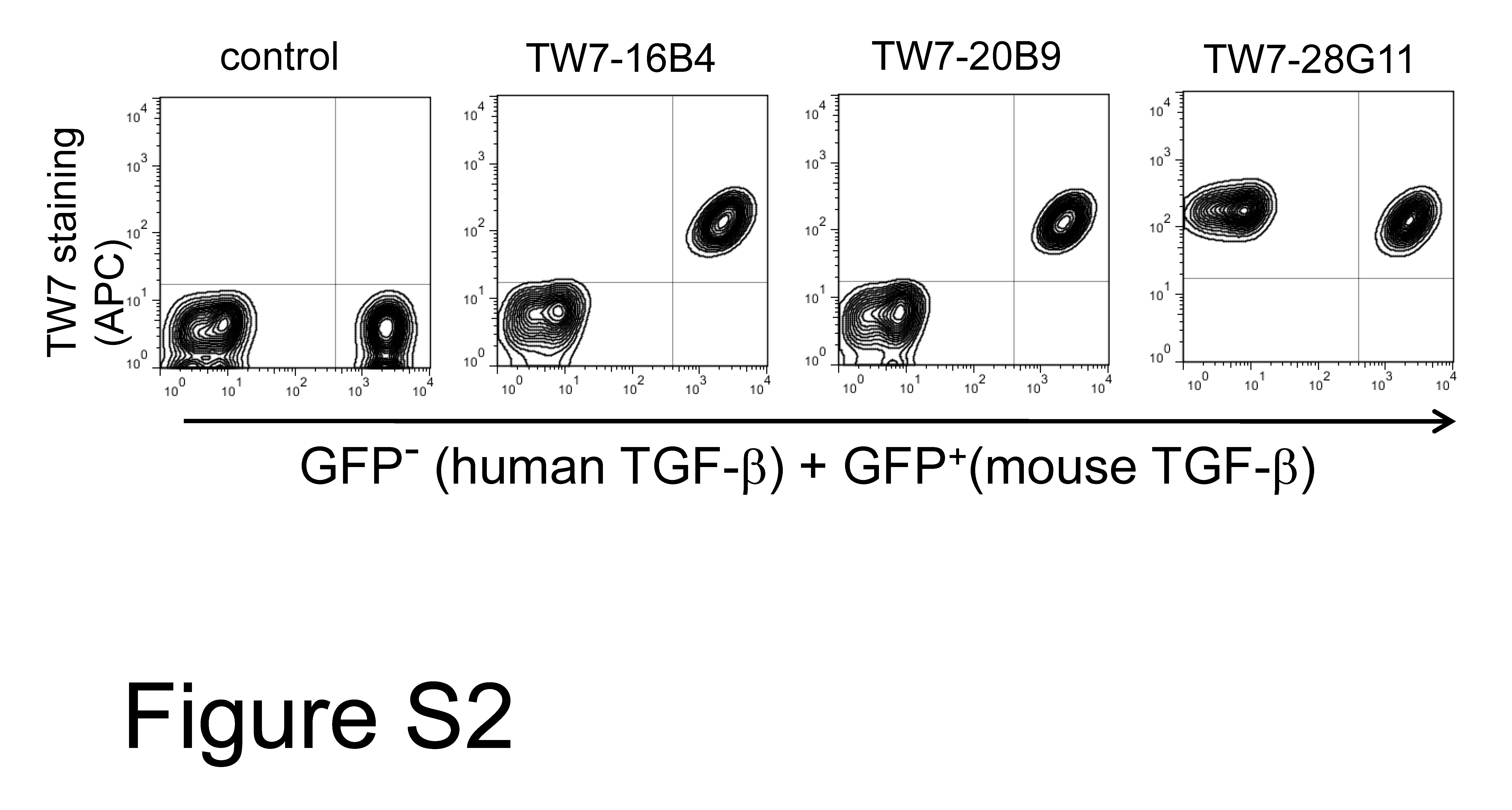

Supplement: Figure S2 — Species specificity of TW7 antibodies. human TGFB1-transduced P3U1 cells (clone 32, without IRES-GFP) (GFP− population) and mouse Tgfb1-transduced P3U1 cells (clone 11, containing IRES-GFP) (GFP+ population) were mixed and surface stained with TW7-16B4, TW7-20B9, or TW7-28G11 mAb. TW7-16B4 and TW7-20B9 stained only mouse Tgfb1-transduced cells while TW7-28G11 stained both human TGFB1-transduced cells and mouse Tgfb1-transduced cells. (TIF) [file pone.0018365.s002.tif]

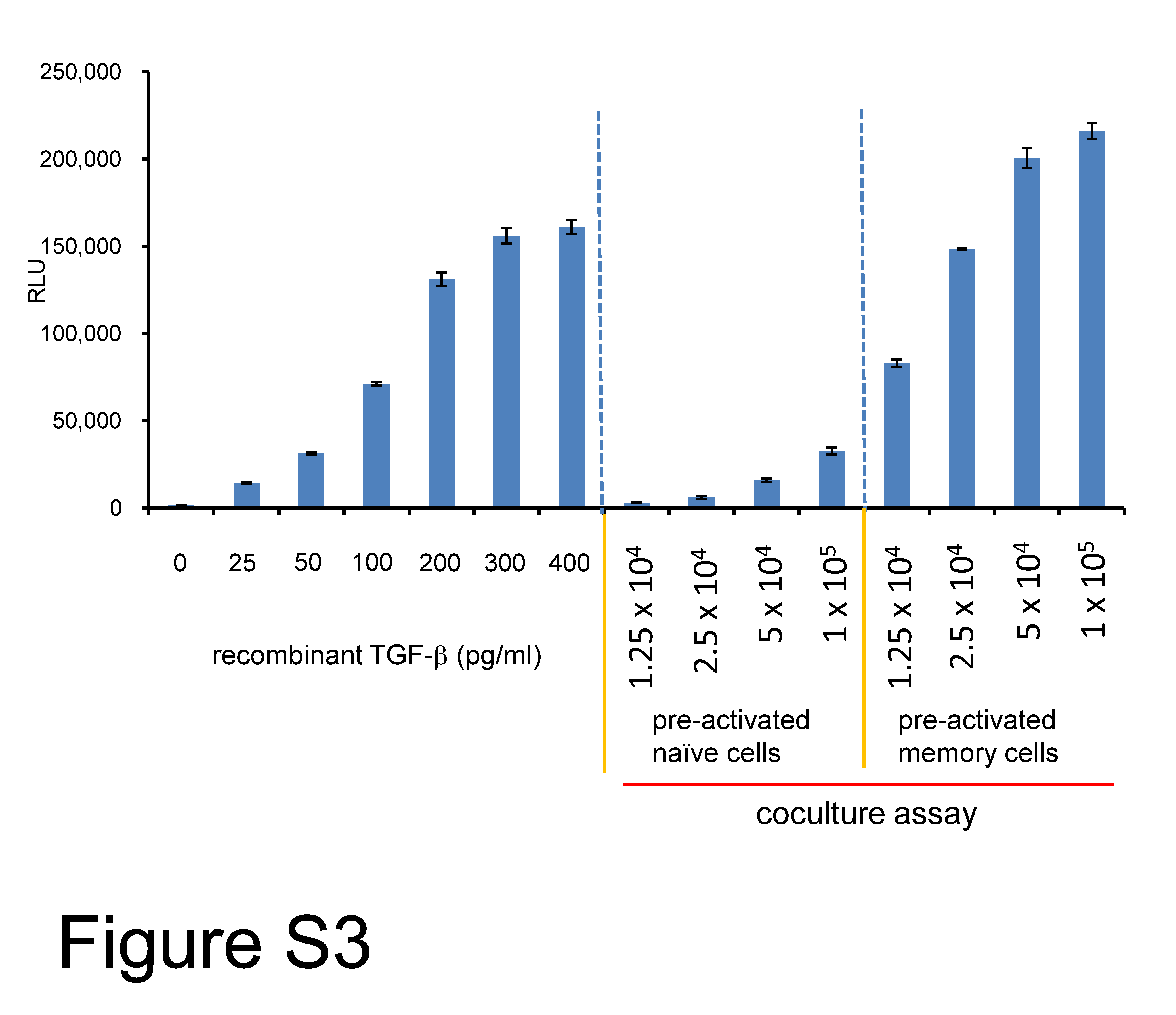

Supplement: Figure S3 — Production of TGF-β activity from naïve CD4 T cells and memory CD4 T cells. CD62L+CD44lo naïve CD4 T cells or CD62LloCD44hi memory CD4 T cells were stimulated with plate-bound anti-CD3/CD28 for 2 days, and rested for 1 day. The pre-activated CD4 T cells were recovered, and the indicated numbers of T cells were added to 293T-caga-Luc-CD32-CD86 cells with 0.5 µg/ml of anti-CD3 antibody. Recombinant human TGF-β was also added as a standard. After 16 hr culture, the reporter cells were lysed and the luciferase activity was measured. Error bars represent mean ± S.D. of duplicates. (TIF) [file pone.0018365.s003.tif]
